# Transcript profiling of a bitter variety of narrow-leafed lupin to discover alkaloid biosynthetic genes

**DOI:** 10.1093/jxb/erx362

**Published:** 2017-11-16

**Authors:** Ting Yang, Istvan Nagy, Davide Mancinotti, Sophie Lisa Otterbach, Trine Bundgaard Andersen, Mohammed Saddik Motawia, Torben Asp, Fernando Geu-Flores

**Affiliations:** 1 Section for Plant Biochemistry, Department of Plant and Environmental Sciences, Faculty of Science, University of Copenhagen, Denmark; 2 Copenhagen Plant Science Centre, Department of Plant and Environmental Sciences, Faculty of Science, University of Copenhagen, Denmark; 3 Section of Crop Genetics and Biotechnology, Department of Molecular Biology and Genetics, Aarhus University, Denmark

**Keywords:** Alkaloid biosynthesis, copper amine oxidase, narrow-leafed lupin, next-generation sequencing, quinolizidine alkaloids, transcript profiling

## Abstract

Lupins (*Lupinus* spp.) are nitrogen-fixing legumes that accumulate toxic alkaloids in their protein-rich beans. These anti-nutritional compounds belong to the family of quinolizidine alkaloids (QAs), which are of interest to the pharmaceutical and chemical industries. To unleash the potential of lupins as protein crops and as sources of QAs, a thorough understanding of the QA pathway is needed. However, only the first enzyme in the pathway, lysine decarboxylase (LDC), is known. Here, we report the transcriptome of a high-QA variety of narrow-leafed lupin (*L. angustifolius*), obtained using eight different tissues and two different sequencing technologies. In addition, we present a list of 33 genes that are closely co-expressed with *LDC* and that represent strong candidates for involvement in lupin alkaloid biosynthesis. One of these genes encodes a copper amine oxidase able to convert the product of LDC, cadaverine, into 1-piperideine, as shown by heterologous expression and enzyme assays. Kinetic analysis revealed a low *K*_M_ value for cadaverine, supporting a role as the second enzyme in the QA pathway. Our transcriptomic data set represents a crucial step towards the discovery of enzymes, transporters, and regulators involved in lupin alkaloid biosynthesis.

## Teaser

We report the first transcript profiling of a high-alkaloid lupin variety and identify a new alkaloid biosynthetic gene, thus accelerating the efforts to develop alkaloid-free lupin crops.

## Introduction

Lupins (*Lupinus* spp.) are minor legume crops that produce beans with a remarkably high protein content (up to 40%). During most of human history, however, they were grown primarily as green manure, as the toxic alkaloids in the beans hindered their use as food and feed crops. In the early 1930s, German and Russian breeders produced the first low-alkaloid varieties directly suitable for human and animal consumption (Ivanov *et al.*, 1932; von Sengbusch, 1930). The introduction of these ‘sweet varieties’ paved the way for wider adoption in Europe and later in Australia, but at the same time resulted in higher susceptibility to herbivores (Wink, 1990; Kozłowski *et al.*, 2016). This is not surprising given the proposed roles of the lupin alkaloids in defense (Wink, 1998). In addition, sweet varieties are not alkaloid-free, and the alkaloid levels in sweet beans vary greatly from year to year under field conditions, often surpassing the thresholds established by the food and feed industries (Cowling and Tarr, 2004). This variation is dependent on complex genotype–environment interactions yet to be unraveled (Cowling and Tarr, 2004).

The toxic alkaloids in lupins belong to the family of quinolizidine alkaloids (QAs), whose common feature is the presence of at least one quinolizidine ring in their chemical structures. Present in legumes of the tribes *Sophoreae* and *Genisteae* (Bunsupa *et al.*, 2012*b*), QAs are being increasingly recognized as a pharmacologically important class of compounds with a wide range of bioactivities, including anti-tumor, anti-viral, and hypoglycemic (Liang *et al.*, 2012; Yang *et al.*, 2012). Interestingly, a particular QA called sparteine has found prominent use in chemistry, where it is employed as a ligand in asymmetric synthesis (Chuzel and Riant, 2005; Firth *et al.*, 2014). The chemical synthesis of sparteine is too complex to be viable and, thus, commercial sparteine is obtained from natural sources. During the last decade, however, sparteine has become much less available for reasons that remain unclear (Ritter, 2017). This has prompted a strong interest in the development of synthetic analogs (O’Brien, 2008).

**Fig. 1.**
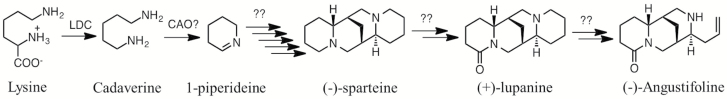
Core pathway towards the tetracyclic QAs in narrow-leafed lupin. LDC, lysine decarboxylase; CAO, cadaverine oxidase.

In order to fully exploit the potential offered by lupins, both as a source of QAs and as a protein crop, there is a need to understand the basic mechanisms behind QA biosynthesis. Lupins accumulate many different QAs, most of which are derivatives of sparteine, which is the simplest tetracyclic QA (Wink *et al.*, 1995) (see structures in Fig. 1). The core biosynthesis of QAs remains a biochemical mystery, as only the first enzyme in the pathway, lysine decarboxylase (LDC), has been discovered so far (Fig. 1) (Bunsupa *et al.*, 2012*a*). The second step has been postulated to be catalyzed by a copper amine oxidase (Bunsupa *et al.*, 2011, 2012*b*), but this proposal awaits experimental validation, including cloning of the respective gene and characterization of the enzyme. What follows next in the pathway, namely the conversion to (–)-sparteine, is a notorious ‘black box’ in plant biochemistry. From (–)-sparteine, the pathway takes different routes depending on the species/variety. In narrow-leafed lupin (*L. angustifolius*, NLL), for example, this route involves oxygenation to give lupanine and subsequent ring opening to give angustifoline. The differently oxygenated QA backbones can be subject to decorations such as glycosylation and acylation (Bunsupa *et al.*, 2012*b*). A dedicated acyltransferase adding tigloyl groups to two related QA backbones has been cloned from white lupin (*L. albus*) (Okada *et al.*, 2005).

In accordance with their status as orphan crops, the available genomics and transcriptomics resources for lupins are limited. Such set of resources is being developed most rapidly for NLL, which is currently the most widely cultivated lupin species. However, the genomic and transcriptomic data obtained so far mainly stem from sweet varieties (Yang *et al.*, 2013; Kamphuis *et al.*, 2015). The lack of tissue-specific transcriptomic data on bitter varieties has hampered the study of QA biosynthesis.

Here, we report the transcriptome of a bitter variety of NLL, obtained using eight different tissues and two different sequencing technologies. In addition, we performed a co-regulation analysis using LDC, which yielded 33 genes that are co-expressed with this gene and that represent strong candidates for enzymes, transporters, and regulators involved in lupin alkaloid biosynthesis. One of these genes encodes a copper amine oxidase able to convert cadaverine into 1-piperideine, as shown by heterologous expression and enzyme assays. Kinetic analysis of this enzyme revealed a low *K*_M_ value for cadaverine, supporting a role in the lupin alkaloid pathway.

## Materials and methods

### Plant materials and RNA extraction

NLL cv. Oskar (bitter, HR Smolice, Poland) was grown in the greenhouse at 22 ± 2 °C under long-day conditions (16 h light/8 h dark). When plants started to set seeds, tissue was harvested from small pods including seeds (~2 cm), large pods not including seeds (~5 cm), large seeds (the seeds inside the large pods), flowers, pedicels, leaves, stems, and roots. For each tissue, total RNA was extracted using the Spectrum™ Plant Total RNA kit (Sigma-Aldrich, USA), and RNA quality and quantity were assessed using a BioAnalyzer (Agilent Technologies, Germany).

### Library construction and sequencing

RNA samples were provided to Macrogen (South Korea) for library construction and sequencing. For short-read sequencing (~125 bp), paired-end TruSeq libraries were prepared and sequenced on a HiSeq2500 instrument (Illumina, USA). For long-read sequencing (>1000 bp), equal amounts of RNA from large pods, large seeds, flowers, pedicels, leaves, and roots were pooled and sequenced using the Pacific Biosciences system (PacBio, USA). For this purpose, four size-specific SMRTbell template libraries were prepared using the pooled RNA (1000–2000, 2000–3000, 3000–6000, and >6000 bp) according to the manufacturer’s instructions. Long-read sequencing was carried out on a PacBio RS II instrument using the P5-C3 chemistry at Macrogen (South Korea).

### Assembly of the transcriptome combining Illumina and PacBio reads

PacBio reads were processed using the Pacific Biosciences IsoSeq™ SMRT (single molecule real time) Analysis software pipeline (v2.3.0, http://www.pacb.com) (Gordon *et al.*, 2015) to produce full-length (FL) high-quality (HQ) transcript isoforms (with parameter Minimum Quiver Accuracy to Classify an Isoform as HQ=0.99, FL Require PolyA=yes).

Raw Illumina reads were first pre-processed to trim adaptors and low quality terminal nucleotides using Scythe (https://github.com/vsbuffalo/scythe) and Cutadapt (Martin, 2011). The trimmed Illumina reads were mapped onto the previously produced PacBio isoforms using the ‘Mirabait’ utility of the MIRA package (Chevreux *et al.*, 2004). The unmapped fraction of the Illumina reads was assembled into contigs using Trinity (v2.1.1) (Grabherr *et al.*, 2011).

Trinity-assembled contigs and PacBio isoforms were combined into a single data set. Chloroplast-specific transcripts were identified by blastn searches against the published chloroplast genome of *L. luteus* (GenBank accession ID: KC695666) and were subsequently removed. rRNA was identified using RNAmmer (Lagesen *et al.*, 2007) and Barrnap (https://github.com/tseemann/barrnap), and was also removed. The remaining sequences were clustered at the 100% similarity level using CD-Hit-EST (Li and Godzik, 2006) to identify short, redundant transcripts. Finally, these redundant transcripts were removed to obtain the bitter NLL transcriptome, which was deposited in NCBI as BioProject PRJNA386115.

### CDS prediction and functional annotation

Coding sequences (CDS) were predicted using GeneMark S-T (Tang *et al.*, 2015) and were translated into proteins. Translated protein sequences were subjected to blastp searches against *Viridiplantae* protein sequences from UniProt (UniProtKB/TrEMBL). An e-value cut-off of 1 × 10^3^ was applied, and top hit sequences were collected for further comparative analyses. A blastp analysis against 62 319 annotated protein sequences from *Medicago truncatula* (Mt4.0v1, http://www.medicagogenome.org) was also performed.

Predictive analysis of conserved protein domains, protein family relationships, signal peptides, transmembrane domains, and Gene Ontology (GO) data were obtained by scanning the InterPro databases via local searches (InterProScan-5.16–55.0) (Jones *et al.*, 2014). Per gene GO term information was collected from the InterProScan outputs using custom scripts.

### Assessment of assembly quality, and completeness

Contig sequences were subjected to reciprocal blastn analysis using a database built from the publicly available transcript assembly of the sweet NLL cultivar Tanjil (https://www.ncbi.nlm.nih.gov/bioproject/PRJNA248164). Assembly completeness and the representation of single-copy ortholog sequences was assesed by BUSCO (Benchmarking Universal Single-Copy Orthologs; Version 2.0) (Simão *et al.*, 2015).

### Gene expression profiling

The abundances of individual transcripts were quantified using Kallisto (Bray *et al.*, 2016). The expression values in transcripts per million (TPM) were log-2 transformed and subjected to hierarchical clustering using MultiExperiment Viewer (Version 4.9.0, http://www.tm4.org). Transcripts were clustered with the self-organizing tree algorithm using Pearson correlation as distance metric. Transcripts with low expression in leaves, stems, or pedicel (TPM <4) were not included in the clustering.

### Quantitative reverse transcription–PCR

The RNA transcript levels of *LDC* and *LaCAO* (encoding *L. angustifolius* copper amine oxidase) were determined by quantitative reverse transcription–PCR (qPCR) analysis as described before (Vandesompele *et al.*, 2002). The RNA samples used were the same samples used for the RNA-seq. Gene-specific primers were designed using Primer3Plus software (https://primer3plus.com/cgi-bin/dev/primer3plus.cgi). For *LDC*, we used primers 5'-ACCGGAACTGGAACTTGATG-3' and 5'-TTGGGGTGGAA TATGCTAGG-3' with an expected product size of 128 bp. For *LaCAO*, we used primers 5'-ACTCACCCGATGAGCTGTTTC-3' and 5'-GGCCAGTCTTCTAAACGAGG-3' with an expected product size of 166 bp. For normalization, we used both *L. angustifolius* 18S rRNA (Fw, 5'-TGGTGCCGGTCTTGCTTAAC-3'; and Rv, 5'-CTACTGGCAGGATCAACCAG-3') and the ubiquitin gene (Fw, 5'-TGACAGCCCACTGAATTGTGAT-3'; and Rv, 5'-TCTTGGGCATAGCAGCAAGC-3'). Average efficiencies of the primers were 1.98 for *LDC*, 2.16 for *LaCAO*, 1.83 for 18S rRNA, and 1.77 for the ubiquitin gene. We measured three technical replicates for every sample.

### Subcellular localization analysis

The full-length cDNA of *LaCAO* was USER-cloned (Nour-Eldin *et al.*, 2010) into two modified pCAMBIA1300 vectors encoding eGFP (enhanced green fluorescent protein) either upstream or downstream of the insertion site (Laursen *et al.*, 2016). The plasmids encoding the peroxisomal or plastidal mCherry markers (mCherry–peroxisome and mChrerry–plastid) (Nelson *et al.*, 2007) were acquired from the Arabidopsis Stock Center (http://www.arabidopsis.org/).

*Agrobacterium tumefaciens* strain LBA4404 virGN54D containing the plasmid of interest was cultured at 28 °C with shaking for 20 h. *Agrobacterium* pellets were collected by centrifugation and then resuspended in the infiltration medium (10 mM MES pH 5.6, 10 mM MgCl_2_, 100 µM acetosyringone) to an OD_600_ of 0.15. Equal volumes of *Agrobacterium* solutions containing a marker gene (mCherry–peroxisome or mCherry–plastid) and a gene of interest (GFP–LaCAO or LaCAO–GFP) were mixed and infiltrated into the abaxial side of *Nicotinana benthamiana* leaves by 1 ml needleless syringes. At 3 d post-infiltration, leaf discs were excised and mounted with water for observation by a SP5x confocal laser scanning microscope equipped with a DM6000 microscope (Leica, Germany). Excitation/emission wavelengths were 488/500–550 for eGFP and 587/598–640 for mCherry. All images were sequentially acquired and processed using the microscope imaging software LAS (version 2.7.3.9723, Leica).

### Chemical synthesis of *N*-methylputrescine

**Fig. 2.**
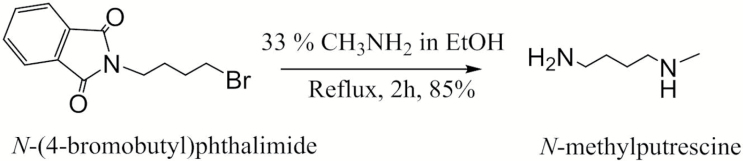
Chemical synthesis of *N*-methylputrescine.

Although the chemical synthesis of *N*-methylputrescine has been reported in the literature, published methods are not straightforward, suffering from a complexity that contrasts with the simplicity of the molecule. We developed a one-step synthesis as shown in Fig. 2.

Our strategy was based on the fact that methylamine in ethanol can be used for the removal of phthalimido-protecting groups from amines in high yield (Motawia *et al.*, 1989). Thus, the reaction between the commercially available *N*-(4-bromobutyl)phthalimide and methylamine resulted in both removal of the phthalimido-protecting group and condensation with methylamine to give *N*-methylputrescine, which was separated as dihydrochloride without chromatographic purification.

*N*-(4-Bromobutyl)phthalimide (1.41 g, 5 mmol) was dissolved in 99.9% ethanol (30.0 ml), and 33% methylamine in absolute ethanol (20 ml) was added. The reaction mixture was heated under reflux for 2 h, after which it was cooled down and evaporated to dryness under reduced pressure. The residue was treated with 1 N HCl (30 ml) and washed with dichloromethane (3 × 30 ml). The aqueous phase was separated, evaporated, and co-evaporated with toluene (3 × 30 ml). The residue was crystallized twice from 99.9% ethanol to afford pure *N*-methylputrescine (0.43 g, 85%) as white needles. The measured melting point (178 ^o^C) and NMR spectra (^1^H and ^13^C) were in agreement with those reported in the literature (Dudley and Thorpe, 1925; Garrido *et al.*, 1984).

### Expression and purification of LaCAO

*LaCAO* (GenBank accession MF152953) was amplified by PCR from *L. angustifolius* cDNA using primers LaCAO-InFusion-For (5'-TTCCAGGGGCCCCTGGCAAT GGCTTCAGCTTCT GAAAAAATG-3') and LaCAO-InFusion-Rev (5'-GTCGACCC GGGAATTTTAGA GCTTTGATGCTAAGGAATTCT-3'). The PCR product was cloned into expression vector pGEX-6P-1 (GE Healthcare) using the In-Fusion HD Cloning Kit (Clontech Laboratories) to give pGEX-6P-LaCAO.

For every round of expression, pGEX-6P-LaCAO was transformed into the *Escherichia coli* strain Rosetta™(DE3)pLysS. A 200 ml culture was grown at 37 °C in selective LB medium up to an OD_600_ of between 0.6 and 0.8. Following induction with 0.2 mM isopropyl-β-d-1-thiogalactopyranoside (IPTG) and addition of 0.1 mM CuSO_4_, the culture was incubated overnight at 28 °C. Cells were harvested and resuspended in 10 ml of phosphate-buffered saline (PBS; pH 7.3). Cell lysis was achieved using a cell disruptor at 30 000 psi, and cell debris was removed by centrifugation at 20 000 *g* for 20 min. The glutathione *S*-transferase (GST)-tagged LaCAO was purified by binding to 400 µl of a suspension of glutathione–Sepharose^®^ 4B for 1 h at 4°C followed by cleavage with GST-tagged PreScission Protease in cleavage buffer (50 mM Tris–HCl, 150 mM NaCl, 1 mM EDTA, pH 7.5) for 2.5–4.0 h at 4°C as recommended by the manufacturer (GE Healthcare). DTT was not added to any of the buffers used for the purification as it was shown to impair the activity of the purified protein. The purification process was monitored by SDS–PAGE, and protein concentrations were measured using the Bradford method.

### Identification of reaction products by GC-MS

One μg of purified LaCAO was added to 400 µl of 500 µM diamine in 50 mM Tris–HCl (pH 8) in a glass vial and incubated overnight at room temperature. The next morning, 10 µl of 10% KCN_(aq)_ were added, and the mixture was incubated at room temperature for 90 min. Following extraction with 150 µl of ethyl acetate, the organic layer was separated and dried over anhydrous Na_2_SO_4_. Assuming a quantitative yield of nitrile from the diamine, 1 Eq of acetic anhydride was added and the reaction was incubated at room temperature for 10 h before GC-MS analysis. As a positive control, the diamine was substituted with 500 µM 2-cyanopiperidine (Aldrich). As a negative control, heat-inactivated LaCAO was used.

The GC-MS analysis was performed on a Shimadzu GCMS-QP2010 system equipped with an EI (electron ionization) source. Separations were performed on an Rxi-17Sil MS column from Restek (20 m×0.18 mm×0.18 µm) using H_2_ as carrier gas at a constant linear velocity of 50 cm s^–1^. A 1 µl aliquot of sample was injected in splitless mode at 250 °C. The initial temperature of the oven was set at 50 °C and held for 1 min. Afterwards the temperature was ramped to 280 °C at a rate of 30 °C min^–1^ and was maintained at 280 °C for 3 min (total run time=11.66 min). MS data acquisition was done using an electron energy of 70 eV in full scan mode (35–500 *m/z*) with a scan interval of 0.15 s.

### Kinetic studies

To determine *K*_M_ values for different diamines, we used a fluorescence-based, peroxidase-coupled assay (Guilbault *et al.*, 1968) in microtiter plate format. In this assay, the H_2_O_2_ produced by LaCAO was used to oxidize 4-hydroxyphenylacetic acid (4-HPAA) quantitatively to a fluorescent compound (λ_ex_=317 nm; λ_em_=414 nm) in the presence of horseradish peroxidase (HRP). Individual reactions were carried out in a final volume of 185 µl containing 50 mM Tris–HCl (pH 8.0), 500 µM 4-HPAA, 10 U ml^–1^ HRP, 1 µg of purified LaCAO, and the desired concentration of diamine (0–800 µM). Assays were started by the addition of protein, and fluorescence was monitored for 10 min at room temperature using a microplate reader (excitation, 317 nm; emission, 414 nm; cut-off, 325 nm). Reactions were carried out in quadruplicate. The whole set of kinetic experiments was performed twice, giving comparable results. Given low yields of protein expression and purification, different enzyme preparations were used for the different substrates. Thus, *V*_max_ values were not compared among substrates.

For the determination of *K*_M_ values, we assumed that the rate of change of fluorescence over time (d*F*/d*t*) was directly proportional to the rate of the LaCAO reaction. This is supported by the fact that increasing the concentration of either 4-HPAA or HRP did not have any measurable effects on the assay results. d*F*/d*t* values were individually determined for the linear part of the fluorescence versus time plot (0–400 s for all diamines) and were corrected by subtracting the average background d*F*/d*t* (measured when no diamine was added). The corrected d*F*/d*t* values were plotted against diamine concentration and non-linear regression was performed using SigmaPlot (Systat Software Inc.).

## Results

### Transcriptome assembly

We performed RNA-seq on eight different tissues of a bitter variety of NLL using two different sequencing technologies. Short-read sequencing (125 bp paired-end) was performed using Illumina technology on a HiSeq2500 sequencer, resulting in raw sequence data ranging from 5.1 Gb to 5.7 Gb per tissue (Table 1). To complement the Illumina data, we performed long-read sequencing (>1000 bp) on an RNA pool using PacBio technology. For this purpose, the RNA pool was fractionated by size, and libraries of four different size ranges were built: 1000–2000; 2000–3000; 3000–6000; and >6000 bp. PacBio sequencing resulted in RNA-seq data ranging from 0.6 Gb to 1.0 Gb per library (Table 1).

**Table 1.** Summary of Illumina and PacBio sequencing data sets

Platform	Tissue	Insert size (bp)	No. of reads	Data size (Gb)	Average read length (bp)
Illumina	Small pod and seed	125	44 857 110	5.7	120
Illumina	Large pod	125	42 084 656	5.3	120
Illumina	Large seed	125	41 636 652	5.2	120
Illumina	Flower	125	40 402 670	5.1	120
Illumina	Pedicel	125	45 148 356	5.7	120
Illumina	Stem	125	44 914 552	5.7	120
Illumina	Leaf	125	44 290 842	5.6	120
Illumina	Root	125	42 756 388	5.4	120
PacBio	Combined tissues	1000–2000	308 616	0.6	1,874
PacBio	Combined tissues	2000–3000	275 912	0.6	2,349
PacBio	Combined tissues	3000–6000	272 782	1.0	3,751
PacBio	Combined tissues	>6000	192 248	0.9	4,872

**Fig. 3.**
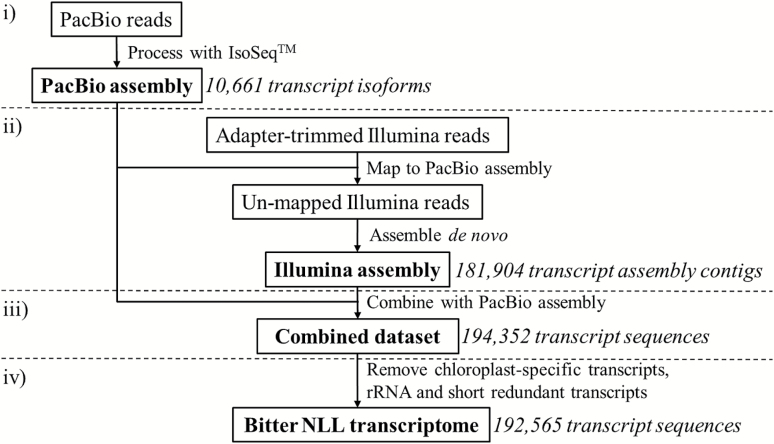
Strategy used to combine the Ilumina and PacBio sequencing results into the bitter NLL transcriptome.

We used a combined assembly strategy to construct the bitter NLL transcriptome (Fig. 3). In the first step, PacBio reads were processed using the IsoSeq™ SMRT pipeline to obtain 10 661 high-quality, full-length transcript isoforms, with the average read length of each library falling within the expected size range (Table 1). In the second step, adaptor-trimmed Illumina reads were mapped to the PacBio isoforms, and 50.8% of them could be mapped accordingly. The unmapped Illumina reads were *de novo* assembled into 181 904 transcript assembly contigs with a median contig length of 487 bp. In the third and final step, the Illumina contigs were added to the PacBio isoforms to give a combined data set of 194 352 transcript sequences. Removal of 439 chloroplast-specific transcripts, 136 rRNA sequences, and 1212 short redundant transcripts resulted in the bitter NLL transcriptome, which contained 192 565 transcript sequences with an N50 transcript length of 1564 bp and a total length of 175.9 Mb (Fig. 3).

### Transcriptome annotation and characterization

Contigs of the bitter NLL transcriptome were subjected to coding sequence prediction using the profile-based approach of the GenMark S-T pipeline (Tang *et al.*, 2015), resulting in the identification of 69 894 coding regions. The respective protein sequences were subjected to blastp searches against all annotated *M. truncatula* reference protein sequences (Mt4.0v1). In this analysis, 99.7% of all bitter NLL protein sequences gave positive hits when an e-value threshold of 1 × 10^3^ was applied, and the percentage of similar amino acids in the top hit alignments was 78% on average. The mean length of queries with hits was 321 amino acids (see Supplementary Fig. S1 at *JXB* online), while the mean length of the queries without hits was 146 amino acids.

The annotations of the top *M. truncatula* BLAST hits were used as the main sources for the annotation of the bitter NLL transcriptome. Furthermore, we used InterPro databases to annotate conserved protein domains, signal peptides, transmembrane domains, and GO terms. Out of 69 894 protein sequences, GO terms were assigned to 38 952 of them (56%). The total number of assigned GO terms was 101 217 (biological process, 30 884; cellular component, 12 551; molecular function, 57 782).

The bitter NLL transcriptome was subjected to BUSCO analysis (v2.0) (Simão *et al.*, 2015) to assess transcriptome completeness based on the representation of near-universal single-copy orthologs selected from OrthoDB. For this analysis, we used the reference ortholog data set embryophyta_odb9, which contains 1440 ortholog sequences from 30 species. BUSCO benchmark results indicated that a high portion of orthologs were represented in our *de novo* assembly, with only 5.6% of the expected orthologs missing (Table 2).

**Table 2.** Results of subjecting the bitter NLL transcriptome to BUSCO benchmark analysis

Complete BUSCOs	1283
Complete and single-copy BUSCOs	767
Complete and duplicated BUSCOs	516
Fragmented BUSCOs	77
Missing BUSCOs	80
Total BUSCO groups searched	1440

We compared our bitter NLL transcriptome with the publicly available transcriptome of the sweet NLL cultivar Tanjil (BioProject PRJNA248164). The Tanjil transcriptome was obtained using Illumina technology and consists of 89 690 contigs, of which 45 739 are predicted to be protein-coding transcripts according to GeneMark S-T predictions. This number represents 65% of the number of protein-coding transcripts in our bitter NLL transcriptome, as predicted using the same analysis (see above). The N50 contig length of the Tanjil transcriptome is 661 bp, which is ~40% of the N50 contig length of our bitter NLL transcriptome (1564 bp).

The two transcriptomes were subjected to reciprocal blastn analyses (see Supplementary Table S1). When transcripts of the bitter NLL transcriptome were used as queries against Tanjil contigs, 61% of queries resulted in hits. When performing blastn in the opposite direction, 97% of Tanjil contigs resulted in hits. From the bitter NLL transcripts without any Tanjil hits, 6251 encoded proteins with blastp hits among *Viridiplantae* species, while the respective number for Tanjil was only 230.

### Selection of candidate genes involved in the biosynthesis of QAs

LDC catalyzes the first step in the QA pathway and is the only known enzyme in the core QA pathway. From the tissues subjected to RNA-seq, the expression level of *LDC* was highest in leaves, stems, and pedicels, with TPM values of 319.0, 233.6, and 202.6, respectively. LDC was expressed at intermediate levels in roots, in small pods including seeds, and in large pods (not including seeds), with TPM values of 17.9, 23.8, and 32.2, respectively. The expression of LDC was not detectable in large seeds and flowers.

**Fig. 4.**
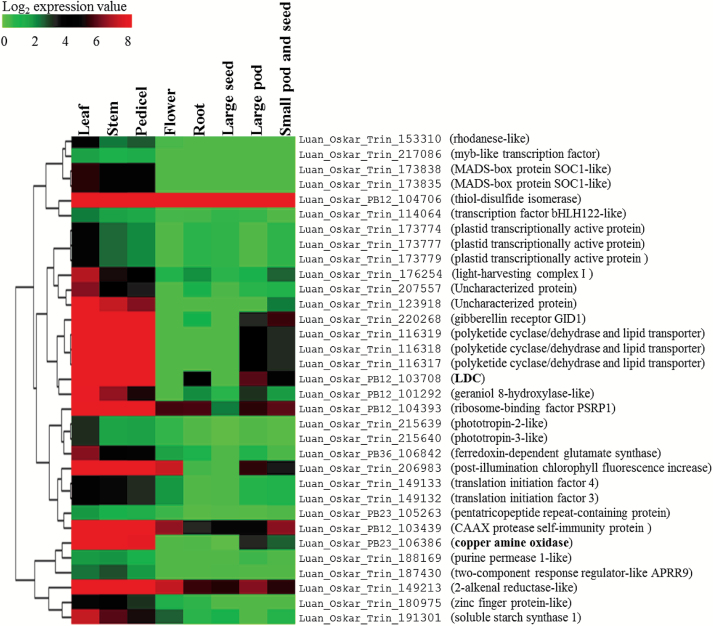
*LDC*-containing transcript cluster selected from the hierarchical clustering analysis of the bitter NLL transcriptome. Columns represent tissues and rows represent transcripts. The color-coding indicates the expression levels of the individual transcripts in the respective tissues. The scale refers to the log_2_-converted TPM values.

In specialized metabolism, genes belonging to the same biosynthetic pathway are usually expressed in a co-ordinated manner (Kim and Buell, 2015). The fact that the tissues that were subjected to RNA-seq presented a wide range of *LDC* expression levels provided an opportunity for the discovery of QA biosynthetic genes via co-expression analysis. Using unsupervised hierarchical clustering, we identified 33 genes with similar expression patterns when compared with *LDC* (Fig. 4).

### Discovery and characterization of cadaverine oxidase

**Fig. 5.**
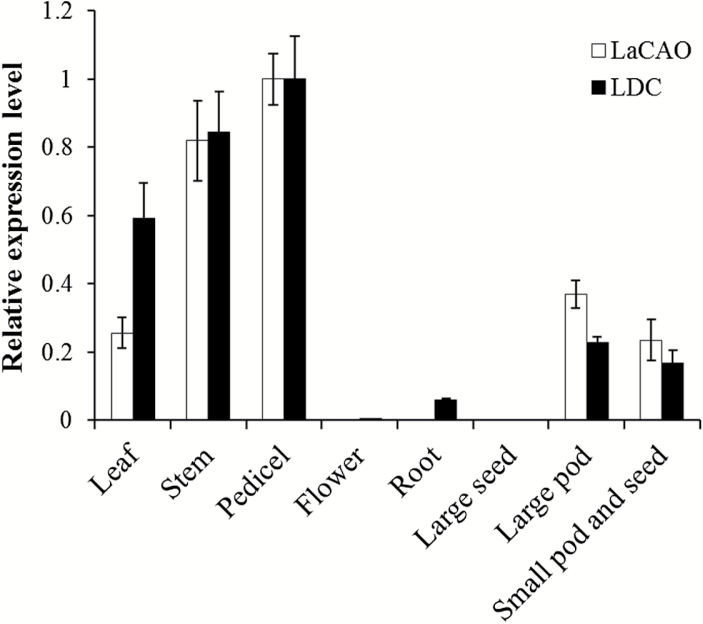
Comparison of the expression patterns of *LaCAO* and *LDC* in different plant tissues. Gene expression levels were determined by qPCR. Expression levels in pedicel were set to 1. Error bars indicate the standard errors from 3 technical replicates.

**Fig. 6.**
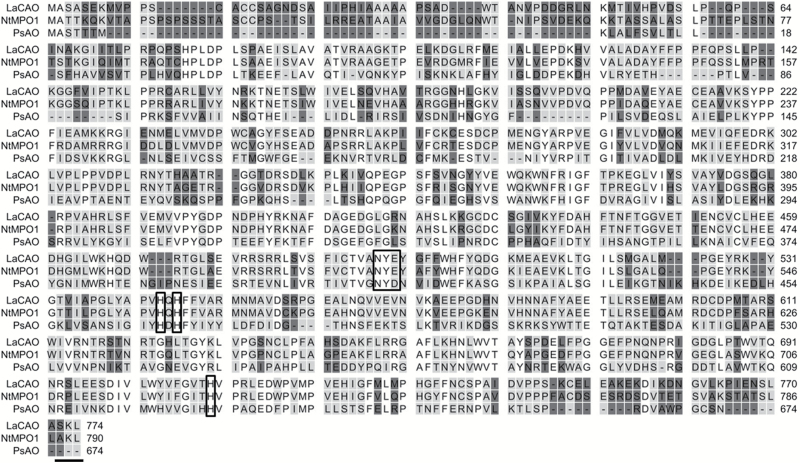
Alignment of cadaverine oxidase from NLL (LaCAO) with two other plant copper amine oxidases, *N*-methylputrescine oxidase from tobacco (NtMPO1) and amine oxidase from pea (PsAO). Amino acid residues that are different between sequences are shaded. The boxes indicate the conserved NYE/X motif and the three histidines interacting with the catalytic copper ion. The horizontal line indicates the peroxisome targeting signal peptide.

**Fig. 7.**
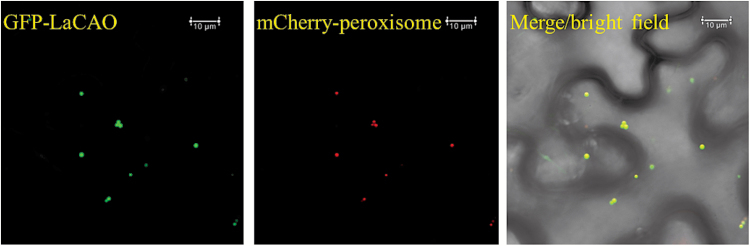
Sub-cellular localization of LaCAO. Confocal images were obtained from leaves of *N. bethamiana* co-expressing an N-terminal GFP fusion (GFP-LaCAO) and the peroxisome-targeted mCherry marker (mCherry-peroxisome). Scale bar = 10 µm.

Among the list of genes closely co-expressed with *LDC* was a gene coding for a copper amine oxidase, which we named *LaCAO* (Luan_Oskar_PB23_106386, see Fig. 1). qPCR confirmed that *LaCAO* showed a similar expression pattern when compared with *LDC* (Fig. 5). We built an alignment of the predicted LaCAO protein with two other copper amine oxidases: *N*-methylputrescine oxidase from *Nicotiana tabacum* (NtMPO1, 75% identity to LaCAO) and amine oxidase from *Pisum sativum* (PsAO, 28% identity to LaCAO). Using this alignment, we identified the highly conserved Asp–Tyr–Glu/X motif (Tanizawa *et al.*, 1994) and the three histidine residues that interact with the catalytic copper ion (Fig. 6). The LaCAO protein sequence also contained a C-terminal SKL tripeptide, which is a typical motif in type 1 peroxisome targeting (Reumann, 2004) (Fig. 6). We examined the subcellular localization of LaCAO by transiently expressing an N-terminal fluorescent protein fusion (GFP–LaCAO) together with a peroxisomal marker (mCherry–peroxisome). The fluorescence of GFP–LaCAO overlapped with the fluorescence of the peroxisomal marker (Fig. 7), indicating that the C-terminus of LaCAO was able to target the protein to peroxisomes.

**Fig. 8.**
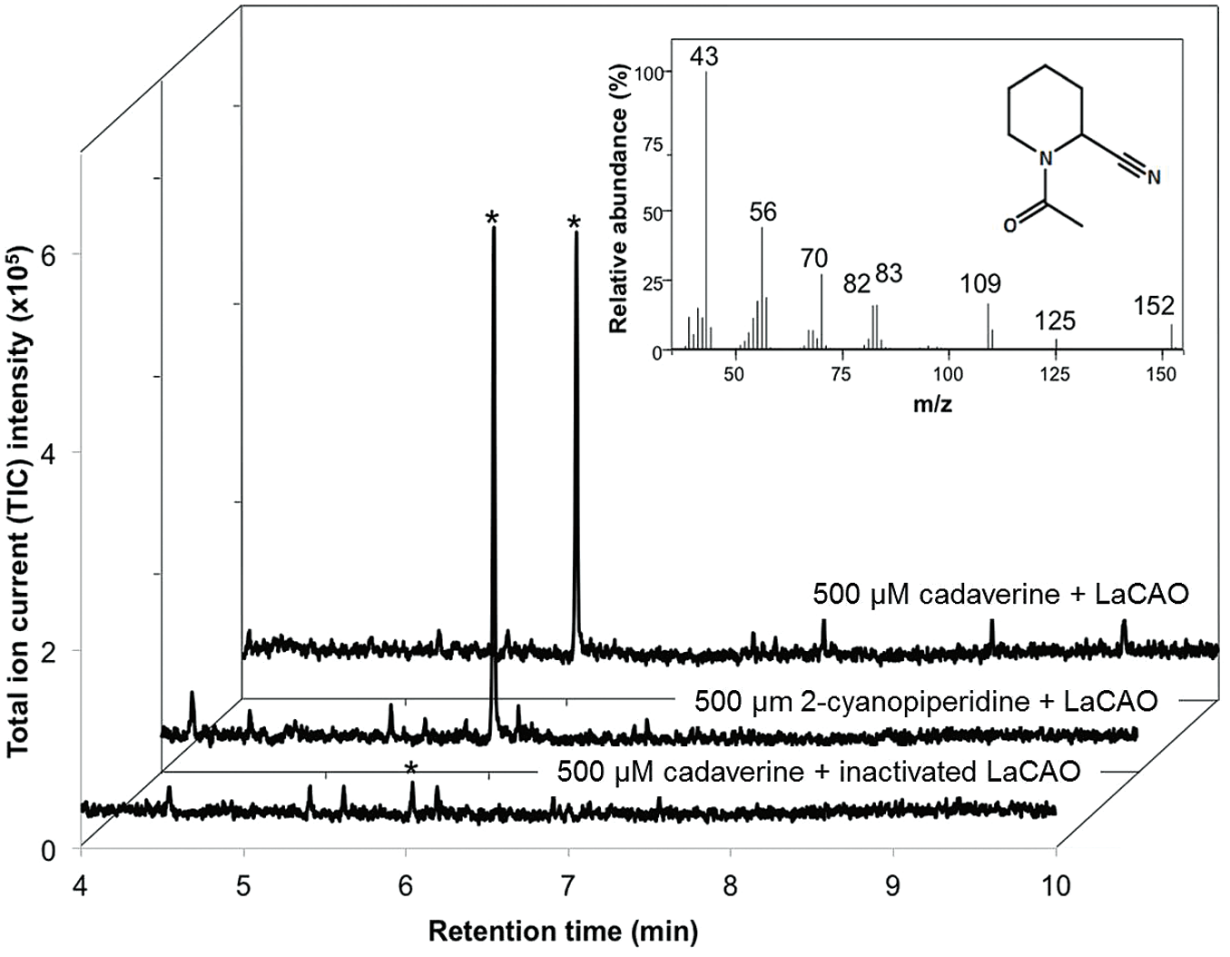
GC-MS analysis of enzyme assays with LaCAO and cadaverine. A chromatogram from an assay with cadaverine (upper chromatogram) is compared to a positive control, where 2-cyanopiperidine was used instead of cadaverine (middle chromatogram). A negative control, where heat-inactivated LaCAO was used instead of active LaCAO, is also shown (lower chromatogram). Reaction products were derivatized before GC-MS analysis. Peaks marked with an asterisk had identical retention times and MS spectra. A representative MS spectrum is shown in the insert. The m/z values of the ion fragments in this spectrum match the ones reported for *N*-acetyl-2-cyanopiperidine (structure within insert) by Tajima and Nakajima (2008).

To characterize the activity of LaCAO, we expressed it in *E. coli* and purified it using GST as a cleavable affinity tag. As expected, the native, purified enzyme oxidized cadaverine into 1-piperideine efficiently. The presence of 1-piperideine was shown by derivatization with cyanide followed by *N*-acetylation to give *N*-acetyl-2-cyanopiperidine (Fig. 8). We also tested the activity of LaCAO against two other diamines, putrescine and *N*-methylputrescine. These two diamines were also oxidized by LaCAO to give the expected products (Supplementary Fig. S2). Furthermore, we studied the kinetics of the LaCAO-catalyzed oxidation of all three diamine substrates using a fluorescence-based, peroxidase-coupled assay. The enzyme displayed the lowest *K*_M_ values for cadaverine and putrescine (6.5 ± 0.5 μM and 4.1 ± 0.2 μM, respectively), while the *K*_M_ value for *N*-methylputrescine was ~5 times higher (20.7 ± 1.6 μM) (Supplementary Fig. S3).

## Discussion

QAs are an important family of plant-derived alkaloids with relevance to the chemical and agricultural industries. However, their core biosynthetic pathway has not been elucidated, and only the first enzyme in the pathway has been discovered. In this study, we report the first tissue-specific transcriptome profiling of a high-QA (bitter) variety of NLL.

Two different sequencing technologies were used, namely Illumina and PacBio. In general, *de novo* transcriptome assembly from Illumina reads can be challenging due to a very large number of short reads and the existence of closely related (but different) transcripts (Martin and Wang, 2011). In order to address this issue, we decided to complement our Illumina data using a long-read (>1000 bp) sequencing technology, PacBio (Bachall, 2009). Prior to the *de novo* assembly of Illumina reads, we first mapped them to the PacBio assembly, and only the remaining unmapped ones were subjected to *de novo* assembly. By doing so, we reduced the workload for the *de novo* assembly of Illumina reads by half and generated a robust and reliable transcriptome, as revealed by BUSCO benchmark analysis and by sequence-level comparison with the reference protein database of the related model species *M. truncatula*. Comparison with a published NLL transcriptome obtained from a sweet variety (Kamphuis *et al.*, 2015) revealed that our transcriptome encoded >6000 additional NLL proteins (Supplementary Table S1) and that the N50 transcript length was more than twice as long. In addition, the RNA-seq data obtained for each tissue in this study (~5 Gb) is more than twice as large as that of the published NLL transcriptome study (~2 Gb), and this probably also resulted in the detection of more transcripts including low expressed genes, gene isoforms, and splice variants.

The tissues used in this study include tissues reported to synthesize QAs, namely leaves (Bunsupa *et al.*, 2012*a*) and stems (Wink, 1987), tissues reported not to be able to synthesize them, namely roots (Wink, 1987), and other tissues with unknown biosynthetic status. Analysis of the expression pattern of *LDC* confirmed the biosynthetic status of leaves and stems, but challenged the claim that roots do not synthesize QAs, as intermediate levels of expression were detected in this organ. With respect to other tissues, it is remarkable that *LDC* expression could not be detected in large seeds, as it has been proposed that half of the high QA content in dry lupin seeds is synthesized *in situ* (Lee *et al.*, 2007). The large seeds in our study were estimated to still be at the filling stage, and thus, absence of *LDC* expression might indicate that QAs in seeds are exclusively transported into the seeds. Detailed studies analyzing *LDC* expression at different stages of seed development are needed to confirm this hypothesis.

Transcriptome profiling followed by gene co-expression analysis is a powerful strategy to identify new genes involved in a given specialized metabolite pathway (Yonekura-Sakakibara *et al.*, 2013). Based on this strategy, we selected a series of genes showing a similar expression pattern to *LDC* (Bunsupa *et al.*, 2012*a*) (Fig. 4). These genes include potential biosynthetic genes such as a geraniol 8-hydroxylase-like gene, regulatory genes such as a myb-like transcription factor, and transporter genes such as a purine permease 1-like transporter. These genes are of great interest for follow-up studies on QA biosynthesis, regulation, and transport in lupin plants. Interestingly, a gene involved in gibberellin signaling (*gibberellin receptor GID1*) also showed a similar expression pattern to *LDC*, indicating that gibberellins may be able to regulate QA biosynthesis as reported for other alkaloids (Pitta-Alvarez and Giulietti, 1997; Srivastava and Srivastava, 2007).

Among the candidate genes selected above, we identified a gene coding for a copper amine oxidase, *LaCAO*. The second step of QA biosynthesis has long been postulated to be catalyzed by a copper amine oxidase converting cadaverine to 5-aminopentanal, which is then spontaneously cyclized to 1-piperideine (Wink and Witte, 1987; Golebiewski and Spenser, 1988; Frick *et al.*, 2017). The presence of a conserved NYE/X motif and three characteristic histidine residues (Kumar *et al.*, 1996) suggested that LaCAO was an active enzyme. We confirmed the activity of LaCAO against cadaverine via heterologous expression, purification, and enzymatic assays, including detection of 1-piperideine via double derivatization. In addition, kinetic experiments showed a high affinity of LaCAO towards cadaverine, with a *K*_M_ of 6.5 ± 0.5 μM. In plants, polyamines such as cadaverine are catabolized by either copper amine oxidases or by FAD-dependent polyamine oxidases as part of poorly understood processes related to cell wall restructuring, programmed cell death, and plant development (Tavladoraki *et al.*, 2016). For these enzymes, the reported *K*_M_ values for cadaverine range from 81 µM to 1919 µM (Equi *et al.*, 1991; Heim *et al.*, 2007; Katoh *et al.*, 2007; Kivirand and Rinken, 2007; Naconsie *et al.*, 2014; Zarei *et al.*, 2015). The low *K*_M_ value obtained for LaCAO, together with its strong co-regulation with LDC, indicates that cadaverine is very likely to be LaCAO’s physiological substrate.

LaCAO localized to peroxisomes (Fig. 7), and this localization contrasts with the subcellular localization of LDC in the plastids (Bunsupa *et al.*, 2012*a*) and with the accumulation of final QA pathway products in vacuoles (Mende and Wink, 1987). Thus, QA biosynthesis might involve frequent subcellular trafficking of pathway intermediates. This possible trafficking of intermediates has also been reported in the biosynthesis of other alkaloids, such as the benzylisoquinoline alkaloids (Beaudoin and Facchini, 2014), the monoterpene indole alkaloids (Payne *et al.*, 2017), and nicotine (Dewey and Xie, 2013). The intracellular transporters involved in these translocation processes may be further engineering targets for the manipulation of QAs and other alkaloids in crop plants.

## Supplementary data

Supplementary data are available at *JXB* online.

Fig. S1. Pairwise length comparison of predicted bitter NLL proteins (queries) and reference *M. truncatula* proteins identified as their top blastp hits.

Fig. S2. MS spectra of the derivatized products from the enzymatic assays with LaCAO against putrescine and against *N*-methyl putrescine.

Fig. S3. Saturation curve of LaCAO against cadaverine, *N*-methylputrescine, and putrescine.

Table S1. Analysis of transcripts without reciprocal blastn hits between our bitter NLL transcriptome and the published Tanjil transcriptome.

## Supplementary Material

Supplementary Material

## Abbreviations:

BUSCO: Benchmarking Universal Single-Copy Orthologs
eGFP: enhanced green fluorescent protein
GO: Gene Ontology
GST: glutathione *S*-transferase
4-HPAA: 4-hydroxyphenylacetic acid
HRP: horseradish peroxidase
IPTG: isopropyl-β-d-1-thiogalactopyranoside
LaCAO: *L. angustifolius* copper amine oxidase
LDC: lysine decarboxylase
NLL: narrow-leafed lupin
QA: quinolizidine alkaloid
qPCR: quantitative reverse transcription–PCR
SMRT: single molecule real time
TPM: transcripts per million.
