# Supplementary material for: Transcript profiling of a bitter variety of narrow-leafed lupin to discover alkaloid biosynthetic genes
Source: J Exp Bot. 2017 Nov 16;68(20):5527–37. doi: 10.1093/jxb/erx362 (PMC5853437; doi:10.1093/jxb/erx362)
Supplement: Supplementary Material [file erx362_suppl_supplementary_material.pdf]

Supplementary data

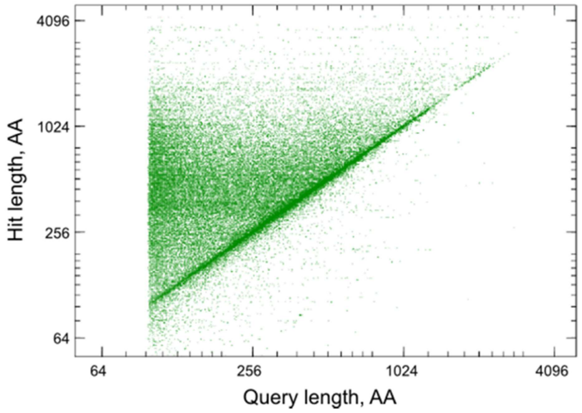

Supplementary Figure 1 Pairwise length comparison of predicted bitter NLL proteins (queries, horizontal) and reference *M. truncatula* proteins identified as their top blastp hits (vertical).

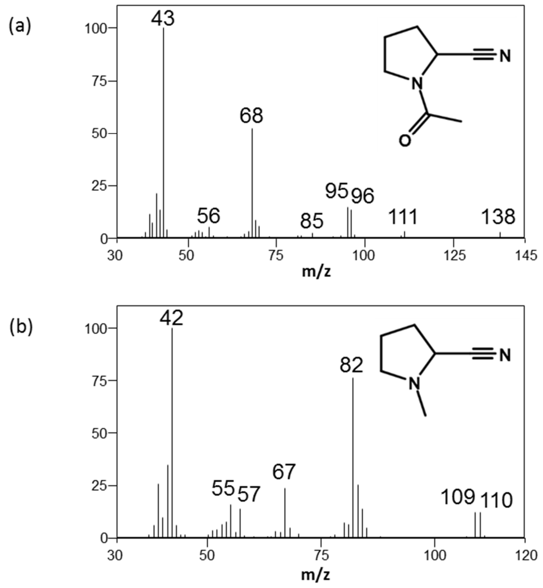

Supplementary Figure 2 MS spectra of the derivatized products from the enzymatic assays with LaCAO against putrescine (a) and against *N*-methyl putrescine (b). Respectively, the expected derivatized products were *N*-acetyl-2-cyanopyrrolidine and *N*-methyl-2-cyanopyrrolidine, and their structures are shown as inserts.

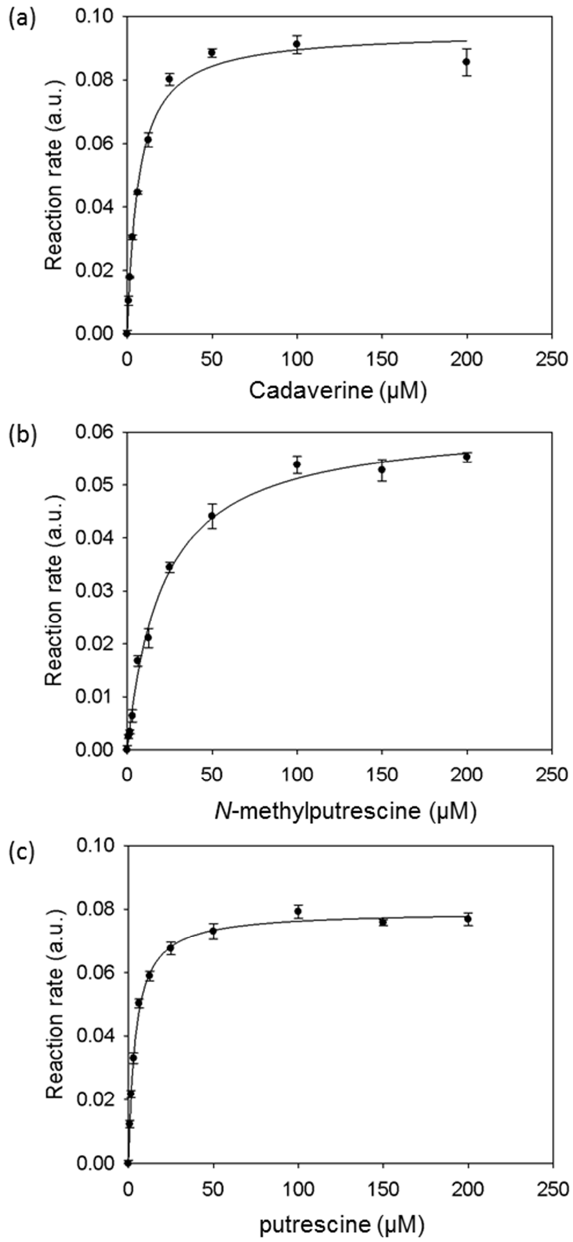

Supplementary Figure 3 Saturation curve of LaCAO against cadaverine (a), *N*-methylputrescine (b) and putrescine (c). Data points represent the mean of four replicates, and error bars represent the respective standard deviations (SD). The curves are the result of non-linear regression fitted to the Michaelis-Menten model of enzyme kinetics.

Supplementary Table 1 Analysis of transcripts without reciprocal blastn hits between our bitter NLL transcriptome and the published Tanjil transcriptome.

	Bitter NLL vs. Tanjil	Tanjil vs. bitter NLL
Total number of transcripts without a blastn hit	75,685	2,900
Number of transcripts with protein-coding sequences	11,613	802
Number of proteins with UniProt/SwissProt blastp hits	9,720	546
Number of proteins with UniProt/SwissProt blastp hits within:		
- Viridiplantae	6,251	230
- Metazoa	3,396	258
- Fungi	25	47
- Bacteria	25	2
- Viruses	11	0
- Unknown	10	9
- Archaea	2	0
